# Sp110 enhances macrophage resistance to *Mycobacterium tuberculosis* via inducing endoplasmic reticulum stress and inhibiting anti-apoptotic factors

**DOI:** 10.18632/oncotarget.19300

**Published:** 2017-07-17

**Authors:** Yongyan Wu, Zekun Guo, Fayang Liu, Kezhen Yao, Mingqing Gao, Yan Luo, Yong Zhang

**Affiliations:** ^1^ Shanxi Key Laboratory of Otorhinolaryngology Head and Neck Cancer, Department of Otolaryngology, Head & Neck Surgery, The First Hospital, Shanxi Medical University, Taiyuan 030001, Shanxi, China; ^2^ College of Veterinary Medicine, Northwest A&F University, Yangling 712100, Shaanxi, China; ^3^ Key Laboratory of Animal Biotechnology, Ministry of Agriculture, Northwest A&F University, Yangling 712100, Shaanxi, China

**Keywords:** apoptosis, endoplasmic reticulum stress, *Mycobacterium tuberculosis*, protein interactome, Sp110

## Abstract

Tuberculosis remains a leading health problem worldwide and still accounts for about 1.3 million deaths annually. Expression of the mouse Sp110 nuclear body protein (Sp110) upregulates the apoptotic pathway, which plays an essential role in enhancing host immunity to *Mycobacterium tuberculosis* (*Mtb*). However, the mechanism of this upregulation is unclear. Here, we have identified 253 proteins in mouse macrophages that interact with Sp110, of which 251 proteins were previously uncharacterized. The results showed that Sp110 interacts with heat shock protein 5 (Hspa5) to activate endoplasmic reticulum (ER) stress-induced apoptosis, and that this is essential for Sp110 enhanced macrophage resistance to *Mtb*. Inhibition of the ER stress pathway abolishing the Sp110-enhanced macrophage apoptosis and resulted in increased intracellular survival of *Mtb* in macrophages overexpressing Sp110 Further studies revealed that Sp110 also interacts with the RNA binding protein, Ncl to promote its degradation. Consequently, the expression of Bcl2, usually stabilized by Ncl, was downregulated in Sp110 overexpressing macrophages. Moreover, overexpression of Sp110 promotes degradation of ribosomal protein Rps3a, resulting in upregulation of the activity of the pro-apoptotic poly (ADP-ribose) polymerase (PARP). In addition, macrophages from transgenic cattle with increased Sp110 expression confirmed that activation of the ER stress response is the main pathway through which Sp110-enhanced macrophages impart resistance to *Mtb*. This work has revealed the mechanism of Sp110 enhanced macrophage apoptosis in response to *Mtb* infection, and provides new insights into the study of host-pathogen interactions.

## INTRODUCTION

Tuberculosis is caused by the intracellular pathogen *Mycobacterium tuberculosis* (*Mtb*) and remains a major human health problem worldwide, infecting a large number of people, making eradication difficult. However, only 10% of latent infections develop into active tuberculosis, indicating that inherited factors play pivotal roles in governing the outcome of *Mtb* infection [1]. Additionally, bovine tuberculosis caused by *Mycobacterium bovis* infection causes substantial economic losses to the cattle industry [2]. Macrophages are the main host cells for *Mtb*, in which infection with *Mtb* induces apoptosis or necrosis and consequently, virulent *Mtb* has evolved to evade the host defense system by inducing necrosis but inhibiting apoptosis [3].

Kramnik *et al*. identified a genetic locus on mouse chromosome 1 that designated susceptibility to tuberculosis 1 (*sst1*), and primarily controls the progression of tuberculosis infection [4]. Subsequent studies identified the intracellular pathogen resistance 1 gene (also known as Sp110) within the *sst1* locus which enhances innate immunity to *Mtb*. Transgenic mouse macrophages overexpressing Sp110 were able to reduce multiplication of *Mtb* efficiently and activate the apoptotic pathway upon infection with virulent *Mtb* [5]. Recently, we reported that overexpression of mouse Sp110 enhances the host's resistance to virulent *Mycobacterium bovis* (*M. bovis*) both *in vivo* and *in vitro* [6, 7]. Furthermore, mutations in the human SP110 gene are associated with immunodeficiency and hepatic veno-occlusive disease [8]. Recent studies focusing on the association of SP110 gene polymorphisms with tuberculosis susceptibility [9–11]. However, the molecular mechanism of Sp110 enhances macrophage resistance to *Mtb* infection remains unknown.

Here, we have characterized the Sp110 protein interaction network with an unbiased proteomic approach, and identified 251 high-confidence, new Sp110-interacting proteins. Subsequent experiments revealed that Sp110 interacts with Hspa5 to activate the ER stress response via sequestering of Hspa5 in the nucleus, thus further inducing mouse macrophage apoptosis. Moreover, our results showed that Sp110 interacts with Ncl and Rps3a to promote their degradation, decreases the stability of the anti-apoptotic B cell leukemia/lymphoma 2 (Bcl2) mRNA to downregulate Bcl2 protein levels, while also upregulating the activity of pro-apoptotic PARP. These results were confirmed by experiments using macrophages from transgenic cattle overexpressing Sp110. Taken together, our data suggest that Sp110 enhances macrophage apoptosis via activating the ER stress response and inhibiting the expression of the anti-apoptotic protein Bcl2.

## RESULTS

### Generation of macrophages expressing biotinylated Sp110

**Figure 1:**
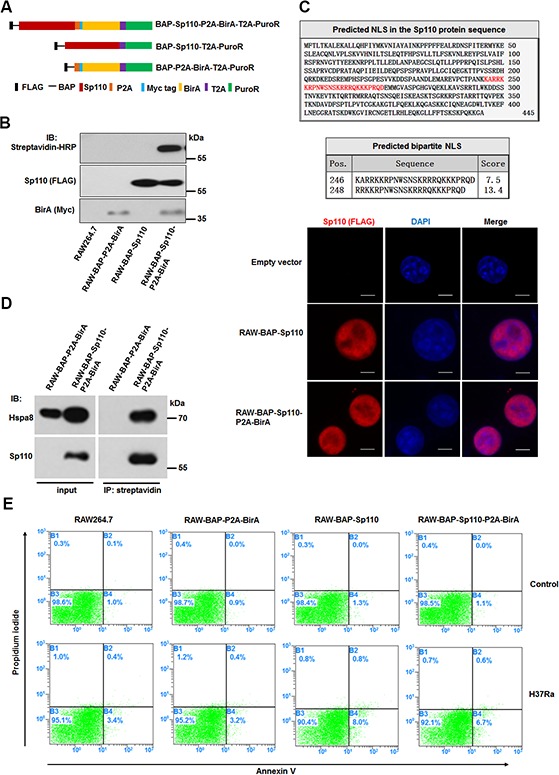
Characterization of macrophages stably expressing biotinylated Sp110 **(A)** The structure of plasmids used for constructing the stably-transfected RAW264.7 cells. **(B)** Verification of biotinylated Sp110. Western blot analysis of the nuclear lysates from the RAW264.7 cells stably transfected with the indicated plasmids. Wild-type RAW264.7 cells were used as a control. **(C)** Bioinformatic prediction and experimental validation of Sp110 nuclear localization. Top: Predicted nuclear localization signal of Sp110 protein. Bottom: Immunofluorescence staining of RAW264.7, RAW-BAP-Sp110, and RAW-BAP-Sp110-P2A-BirA cells with an anti-Flag antibody. Nuclei were stained with DAPI. Scale bar = 5 μm. **(D)** Validation of the interaction between biotinylated Sp110 and Hspa8. IP of the nuclear lysates prepared from RAW-BAP-Sp110-P2A-BirA or RAW-BAP-P2A-BirA cells using streptavidin-conjugated agarose resin. The presence of Sp110 and Hspa8 was detected by immunoblotting. **(E)** Flow cytometric analysis of the apoptosis of RAW264.7, RAW-BAP-P2A-BirA, RAW-BAP-Sp110, and RAW-BAP-Sp110-P2A-BirA cells. The cells were infected with H37Ra at an MOI of 5 for 24 h. Uninfected cells were used as controls.

To isolate the Sp110-interacting proteins, we generated macrophages stably expressing a biotin-tagged Sp110 protein using RAW264.7 cells. We constructed a multi-cistronic lentiviral vector encoding a Flag tag, followed by a biotin acceptor peptide (BAP) fused to the N terminus of the Sp110 protein, along with the *Escherichia coli* biotin ligase BirA separated by a self-cleaving 2A peptide sequence derived from porcine teschovirus-1 (P2A). Constructs encoding BAP-Sp110 alone and the BirA enzyme alone were used as controls (Figure 1A). RAW264.7 cells were transduced with these lentiviral constructs, and puromycin was used to select those that produced stably transduced cells. Subsequently, the biotinylated Sp110 protein was detected only in RAW264.7 cells expressing both the BAP-Sp110 and BirA enzyme simultaneously (RAW-BAP-Sp110-P2A-BirA). No biotinylated protein was detected in cells expressing either BAP-Sp110 alone or the BirA enzyme alone (Figure 1B). These results demonstrate the production of RAW264.7 cells that stably express biotinylated Sp110.

Bioinformatics analysis using the cNLS Mapper (http://nls-mapper.iab.keio.ac.jp/cgi-bin/NLS_Mapper_form.cgi) identified a putative bipartite nuclear localization signal (NLS) within aa 246 to aa 270 of the Sp110 protein sequence. To investigate the effect of biotinylation on the subcellular localization of the Sp110 protein, we determined the distribution of unbiotinylated and biotinylated Sp110 within the RAW264.7 cells by immunofluorescence microscopy. As shown in Figure 1C, both unbiotinylated and biotinylated Sp110 showed nuclear accumulation. Furthermore, immunoprecipitation (IP) results demonstrated that biotinylation does not impair the interaction of Sp110 and its known protein partner Hspa8 (Figure 1D). Lastly, to assess the effect of biotinylation on Sp110 function, the relative levels of cell death of RAW-BAP-Sp110-P2A-BirA and RAW-BAP-Sp110 cells in response to *Mtb* infection was compared. RAW-BAP-Sp110 and RAW-BAP-Sp110-P2A-BirA cells exhibited higher apoptotic rates than the control cells 24 h post-infection with the attenuated strain of *Mtb* H37Ra (Figure 1E). Overall, these data show that biotinylated Sp110 functions similarly to unmodified Sp110.

### Characterization of the Sp110 protein interactome

**Figure 2:**
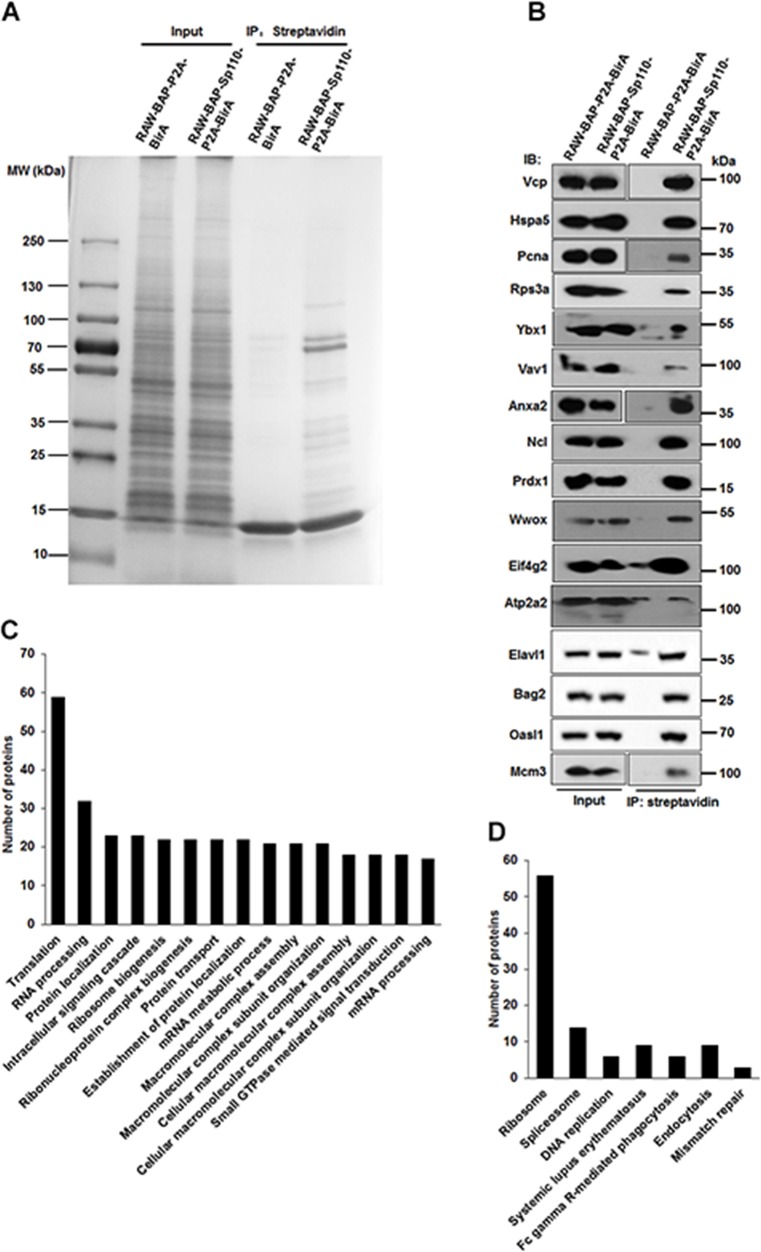
Characterization of the Sp110 protein interactome by affinity purification and mass spectrometry **(A)** Separation of the affinity-purified Sp110 protein complexes using SDS-PAGE followed by Coomassie staining. Data are representative of four experiments. **(B)** Validation of the interactions between Sp110 and identified Sp110-interacting proteins. IP was performed using streptavidin-conjugated agarose resin. The presence of the Sp110 protein partner was detected by immunoblotting using specific antibodies. Data are representative of three experiments. **(C)** GO annotation of Sp110-interacting proteins. The top 15 GO terms (biological process) ranked according to the protein counts are plotted. **(D)** KEGG pathway analysis of Sp110-interacting proteins.

After confirming that the interaction with HSpa8, subcellular localization, and function of biotinylated Sp110 was unchanged compared to unbiotinylated protein, RAW-BAP-Sp110-P2A-BirA cells were used to characterize the Sp110 protein interactome. Proteins complexes containing biotinylated-Sp110 protein were isolated from nuclear extracts of RAW-BAP-Sp110-P2A-BirA cells by streptavidin-mediated affinity purification. Nuclear extracts from RAW-BAP-P2A-BirA cells were used as negative controls. After purification, the protein complexes were separated by SDS polyacrylamide gel electrophoresis (SDS-PAGE). As expected, biotinylated Sp110 bound many proteins of varying molecular weights, with few protein bands observed in the negative control lane (Figure 2A). Individual protein bands were excised, in-gel digested with trypsin, and then identified by high-performance liquid chromatography-electrospray tandem mass spectrometry sequencing. A total of 253 proteins (excluding the bait protein Sp110) were identified. These were repeatedly detected in at least three of four independent experiments in RAW-BAP-Sp110-P2A-BirA cells, but not detected or found at relatively low abundance in RAW-BAP-P2A-BirA cells (Supplementary Table 1). As expected from studies, Hspa8 and Mybbp1a were identified within Sp110 interactome [12, 13]. Interactions of Sp110 with Vcp, Hspa5, Pcna, Rps3a, Ybx1, Vav1, Anxa2, Ncl, Prdx1, Wwox, Eif4g2, Atp2a2, Elavl1, Bag2, Oasl1, and Mcm3 were confirmed by IP and immunoblots (Figure 2B).

To better understand the functions and signaling pathways of the Sp110-interacting proteins, we used the DAVID software package to classify all the identified proteins according to their previously defined function and biological processes. The substantial enrichment observed in proteins that regulate the processes of translation, ribosome biogenesis, RNA processing, mRNA metabolic process, protein localization, and DNA packaging indicated the existence of several unexplored functions for Sp110 (Figure 2C and Supplementary Table 2). Overall, about 35% of the proteins identified as interacting with Sp110 were structural constituents of ribosomes or involved in ribosome biogenesis and protein synthesis (Supplementary Table 3). Further evaluation using the KEGG pathway analysis revealed that the Sp110 interactome was also enriched in proteins associated with the Ribosome, spliceosome, DNA replication, Fc gamma R-mediated phagocytosis, and endocytosis pathways (Figure 2D).

Mass spectrometric sequencing data remarkably revealed that several stress response proteins, in addition to Hspa8, were also candidate Sp110 partners, including Hspa1l, Hsp90b1, Hspa2, and Hspa5 (also known as Grp78 or Bip). The interaction between Hspa5 and Sp110 was confirmed by co-IP (Figure 2B). A network map of the identified Sp110 interactome was constructed using STRING software [14] and, as expected from the preliminary analysis, showed clusters associated with ribosome biogenesis, RNA processing, DNA replication, protein transport, phagocytosis, and stress response (Supplementary Figure 1). These results confirm that we successfully isolated the Sp110 protein complexes from RAW264.7 cells and identified the constituent proteins. Our interactome data suggest that Sp110 likely executes its *Mtb* protective activity by regulating protein synthesis and stress response.

### Sp110 induces ER stress response via sequestering Hspa5 in the nucleus

**Figure 3:**
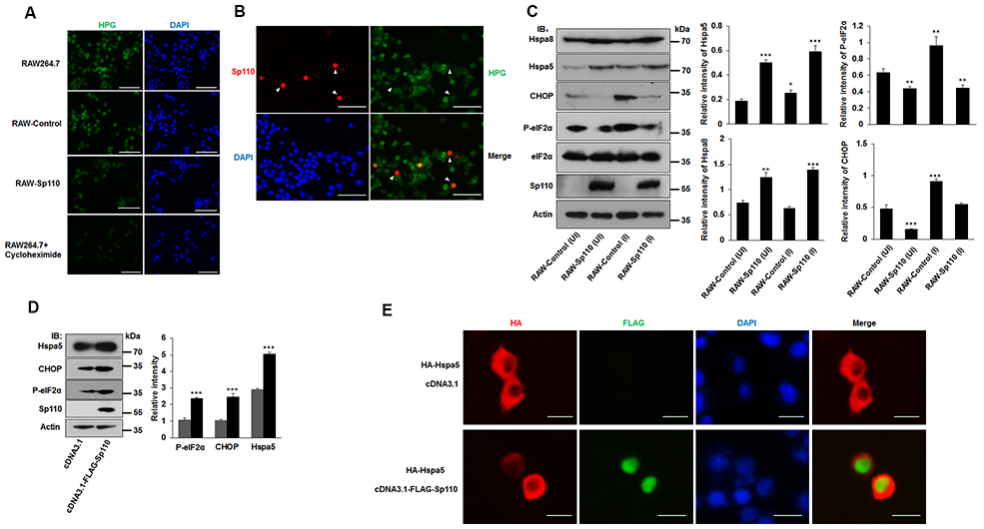
Sp110 induces ER stress responses in mouse macrophages **(A)** The nascent protein synthesis in RAW264.7, RAW-Control and RAW-Sp110 cells were determined by HPG analysis. RAW264.7 cells treated with protein synthesis inhibitor cycloheximide (10 μg/ml, 12 h) were used as positive control. **(B)** RAW264.7 cells were transfected with the Sp110 expression plasmid for 24 h. Sp110 protein was detected by immunofluorescence staining, and protein synthesis was determined by HPG analysis. Nuclei were stained with DAPI. Scale bar = 50 μm. **(C)** Cell lysates prepared from the uninfected (UI) and H37Ra-infected ((MOI 5:1) RAW-Control and RAW-Sp110 cells (I) were analyzed by immunoblotting using antibodies against Hspa8, Hspa5, CHOP, P-eIF2α, eIF2α, Sp110, and Actin. The bands corresponding to Hspa8, Hspa5, CHOP, and P-eIF2α were quantified with ImageJ, and the intensity of each protein was normalized to the intensity of Actin. **(D)** RAW264.7 cells were cotransfected with pcDNA3.1 or pcDNA3.1-Flag-Sp110 for 48 h, the protein levels of Sp110, Hspa5, CHOP, and P-eIF2α were determined by immunoblotting. The bands corresponding to Hspa5, CHOP and P-eIF2α were quantified with ImageJ, and the intensity of each protein was normalized to the intensity of Actin. **(E)** RAW264.7 cells were cotransfected with pcDNA3.1 or pcDNA3.1-Flag-Sp110 and pCMV-HA-Hspa5 for 24 h, and then the subcellular localizations of Sp110 and Hspa5 were examined by immunofluorescence staining. Nuclei were stained with DAPI. Scale bar = 10 μm. Data are present as means ±SD of three independent experiments, * p< 0.05, **p< 0.01, and *** p< 0.001.

The interactome data suggest that Sp110 is involved in the cellular stress process, and recent studies have suggested that *Mtb* can induce ER stress-mediated apoptosis [15, 16]. To investigate the role of Sp110 in ER stress, RAW264.7 cells stably expressing Sp110 (RAW-Sp110) and an empty vector transduced control (RAW-Control) were generated. We found that global protein synthesis was significantly repressed in RAW-Sp110 cells compared with that of control cells (Figure 3A) in agreement with the repression of protein translation previously observed in cells in response to ER stress [17]. This result was also observed in RAW264.7 cells transiently transfected with a Sp110 expression plasmid (Figure 3B). These results suggest that overexpression of Sp110 induces the ER stress response.

Hspa5 is the most commonly used marker of the unfolded protein response (UPR) in response to ER stress, and activation of the UPR also leads to the induction of the transcription factor, C/EBP homologous protein (CHOP), and phosphorylation of eIF2α (P-eIF2α) [18]. Infection of RAW-Control cells with H37Ra increased the levels of Hspa5, P-eIF2α, and CHOP, when compared with uninfected counterparts (Figure 3C). Remarkably, the expression of chaperone proteins Hspa5 and Hspa8 were also significantly increased in both uninfected and *Mtb*-infected RAW-Sp110 cells (Figure 3C). Unexpectedly, levels of P-eIF2α and CHOP proteins were decreased significantly in RAW-Sp110 cells when compared with RAW-Control cells (Figure 3C). This phenomenon is contradictory to the classic UPR, but is consistent with previously reported adaptations to the ER stress response [19]. Furthermore, transient transfection of RAW264.7 cells with the Sp110 expression plasmid increased the protein levels of ER stress markers Hspa5, CHOP and P-eIF2α (Figure 3D), supporting the conclusion that Sp110 induces an ER stress response.

Because Sp110 localizes in the nucleus and interacts with Hspa5, we speculated that Sp110 may change the subcellular localization of Hspa5 to induce the ER stress response. To test this, we examined the subcellular localization of Hspa5 in the presence or absence of Sp110. Our results showed that Hspa5 is found mainly in the cytoplasm, however, upon expression of Sp110, Hspa5 showed increased nuclear accumulation (Figure 3E). Collectively, these data indicate that Sp110 induces ER stress and activates the UPR signaling pathway through its interaction with Hspa5 and sequestering this complex in the nucleus.

### Activation of the ER stress response is essential for Sp110-mediated macrophage resistance to *Mtb*

**Figure 4:**
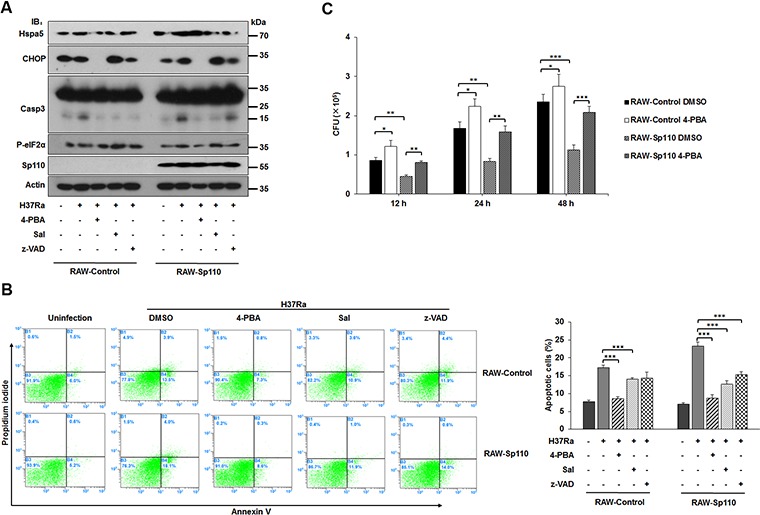
Effect of ER stress response on Sp110-mediated macrophage resistance to *Mtb* **(A)** Expression of ER stress and apoptotic markers of RAW-Control and RAW-Sp110 cells in response to *Mtb* infection and chemical inhibitors. RAW-Control and RAW-Sp110 cells were pretreated with 4-PBA, Sal, or z-VAD-FMK for 1 h, and then infected with H37Ra (MOI 5:1) for 24 h. Protein levels of CHOP, Hspa5, Casp9, Casp3, P-eIF2α, and Sp110 were examined by immunoblotting. **(B)** RAW-Control and RAW-Sp110 cells were pretreated with 4-PBA, Sal, or z-VAD-FMK for 1 h, and then infected with H37Ra (MOI 5:1) for 24 h. Cell apoptosis was quantified by Annexin-V staining followed by flow cytometric analysis. **(C)** Effect of Sp110-mediated ER stress response on intracellular survival of *Mtb*. RAW-Control and RAW-Sp110 cells were pretreated with 4-PBA for 1 h and then infected with H37Ra (MOI 5:1) for 4 h. 4-PBA remained for the rest of the infection. The cells were collected at 12 h, 24 h and 48 h post infection with *Mtb*, and bacteria number was determined by CFU counting. Data are present as means ±SD of three independent experiments,* p< 0.05, **p< 0.01, and *** p< 0.001.

To assess the role of the ER stress response in Sp110-enhanced macrophage apoptosis, the RAW-Control and RAW-Sp110 cells were treated, prior to *Mtb* infection, with the chemical chaperone 4-phenyl butyric acid (4-PBA). As expected, levels of both Hspa5 and CHOP proteins were significantly attenuated, along with a reduction in Casp3 activation for either RAW-Control or RAW-Sp110 cells in this experiment (Figure 4A). Salubrinal (Sal) is a selective inhibitor of eIF2α dephosphorylation that was previously found to protect against ER stress-mediated apoptosis [20]. Sal-treated RAW-Control and RAW-Sp110 cells showed reduced activation of Casp3 after *Mtb* infection compared with their untreated counterparts, although P-eIF2α and CHOP levels were increased by Sal treatment (Figure 4A). Additionally, we investigated the relationship between Sp110-mediated apoptosis and caspase-dependent apoptosis after *Mtb* infection. In both RAW-Control and RAW-Sp110 cells, *Mtb*-induced Casp3 activation was attenuated significantly by caspase inhibitor z-VAD-FMK. These data suggest that induction of ER stress is critical for activation of caspase cascades by Sp110.

To confirm that inhibition of the ER stress response can reduce Sp110-enhanced macrophage apoptosis, we examined the apoptotic rates of *Mtb*-infected RAW-Control and RAW-Sp110 cells in the presence or absence of an ER stress inhibitor. The *Mtb*-induced apoptosis of RAW-Sp110 cells was higher than that of RAW-Control cells, while 4-PBA and Sal treatment both significantly reduced the percentage of apoptotic cells, not only in RAW-Control but also in RAW-Sp110 cells (Figure 4B). Furthermore, inhibition of caspase activation also reduced the level of apoptosis observed in *Mtb*-infected RAW-Control and RAW-Sp110 cells (Figure 4B). Notably, ER stress inhibitors did not completely abolish Sp110-induced apoptosis after *Mtb* infection (Figure 4B), indicating that other Sp110-regulated factors might contribute to Sp110-induced apoptosis. To further investigate whether the Sp110-activated ER stress pathway is involved in intracellular survival of mycobacteria, we assessed the effects of 4-PBA on the intracellular survival rates of *Mtb* in either RAW-Control or RAW-Sp110 cells. As expected, intracellular survival of *Mtb* in RAW-Sp110 was significantly decreased (Figure 4C). Interestingly, intracellular survival of *Mtb* was increased in 4-PBA treated RAW-Control and RAW-Sp110 cells (Figure 4C). Taken together, these data provide evidence that activation of the ER stress response is essential for Sp110-mediated macrophage resistance to *Mtb* infection through induction of apoptosis.

### The interaction between Sp110 and Hspa5 is critical for Sp110-enhanced macrophage apoptosis

**Figure 5:**
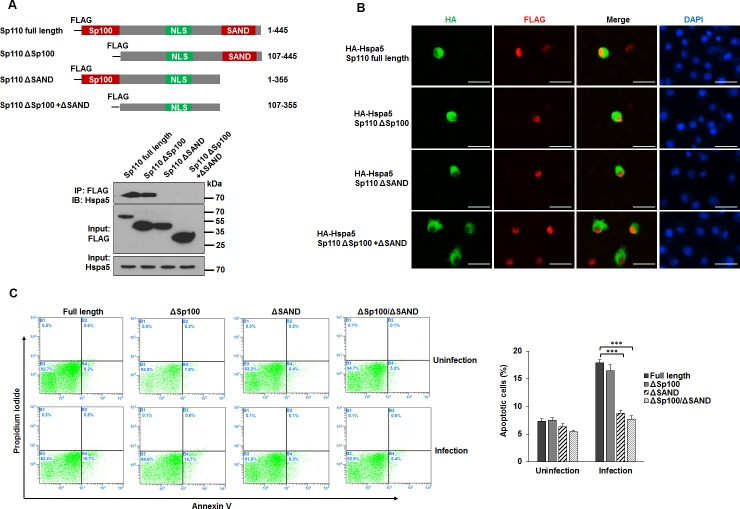
The effect of the interaction between Sp110 and Hspa5 on Sp110 enhances macrophage apoptosis **(A)** Schematic illustration of RAW264.7 cells expressing full-length Sp110 or mutants (top panel). Lysates from RAW264.7 cells expressing the Sp110 mutants were subjected to IP (bottom panel). **(B)** RAW264.7 cells were cotransfected with full-length Sp110 or mutants plus pCMV-HA-Hspa5 for 24 h, and then the subcellular localizations of Sp110 and Hspa5 were examined by immunofluorescence staining. Nuclei were stained with DAPI. Scale bar = 25 μm. **(C)** RAW264.7 cells expressing full-length Sp110 or mutants were infected with H37Ra (MOI 5:1) for 24h. Cell apoptosis was quantified by Annexin-V staining followed by flow cytometric analysis.

Sp110 consists of a Sp100 domain (residues 1-106), an NLS domain (residues 246–270), and a SAND domain (residues 353-433) (Figure 5A, top). To define which domain of Sp110 contributes to the interaction between Sp110 and Hspa5, we constructed RAW264.7 cells expressing domain deletion mutants of Sp110. IP and immunoblots experiments revealed that the SAND domain is required for the interaction between Sp110 and Hspa5 (Figure 5A, bottom). Nuclear localization experiments showed while all Sp110 mutants accumulated in the nucleus, Hspa5 accumulated in the nuclei of cells expressing either full-length Sp110 or the SAND domain to much higher levels than in cells expressing the Sp110 mutant lacking the SAND domain (Figure 5B). Finally, we examined the effects of these Sp110 mutants on apoptosis of RAW264.7 cells after *Mtb* infection. Compared with cells expressing full-length Sp110, cells expressing Sp110 mutants showed lower levels of apoptosis after *Mtb* infection (Figure 5C). In particular, the apoptosis rates in *Mtb*-infected cells expressing the SAND domain deleted Sp110 were similar to their uninfected counterparts (Figure 5C). Thus, promotion of apoptosis in mouse macrophages by Sp110 depends, at least in part, on the interaction of Sp110 and Hspa5, through the SAND domain of Sp110.

### Sp110 interacts with Ncl and Rps3a to enhance the apoptotic pathway

**Figure 6:**
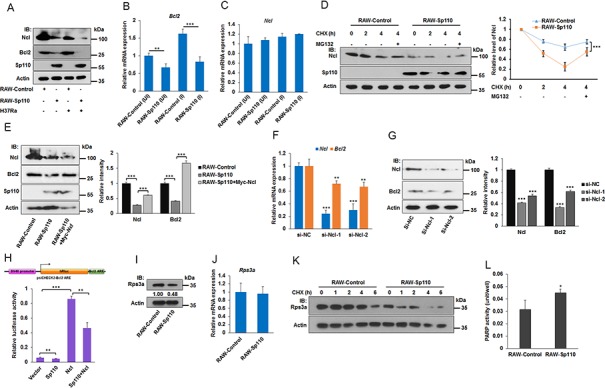
Sp110 promotes Ncl and Rps3a degradation **(A)** Overexpression of Sp110 in RAW264.7 cells decreased the protein levels of Ncl and Bcl2. The protein levels of Sp110, Ncl and Bcl2 in Uninfected and H37Ra infected (MOI 5:1, 24 h) RAW-Control or RAW-Sp110 cells were determined by immunoblotting. **(B)** and **(C)** RAW-Control and RAW-Sp110 cells were infected with H37Ra (MOI 5:1, 24 h), and uninfected cells were used as controls. Expression of Bcl2 and Ncl was monitored by qPCR. **(D)** Sp110 promotes Ncl protein degradation. RAW-Control and RAW-Sp110 cells were treated with protein synthesis Cycloheximide (10 μg/ml) or proteasome inhibitor MG132 (10 μM) for different periods of time. Expression changes of Ncl were determined by immunoblotting. The intensity of Ncl bands was quantified with ImageJ and normalized to the intensity of Actin. **(E)** Sp110 inhibits Bcl2 expression via downregulating Ncl. Expression of Ncl and Bcl2 in RAW-Control, empty vector transfected RAW-Sp110, and pCMV-Myc-*Ncl* transfected RAW-Sp110 cells was monitored by immunoblotting. **(F)** and **(G)** Knockdown of *Ncl* leads to the downregulation of both mRNA and protein levels of the Bcl2. RAW264.7 cells were transfected with siRNAs targeting *Ncl* or negative control siRNA for 48 h, and then the mRNA and protein expression of Ncl and Bcl2 was determined by qPCR and immunoblotting respectively. **(H)** Sp110 decreases the stability of the reporter gene that contains the ARE sequence of Bcl2 mRNA. RAW264.7 cells were cotransfected with psiCHECK2-*Bcl2* ARE and pCDNA3.1-FLAG-*Sp110* or pCMV-Myc-*Ncl* for 48 h followed by luciferase assays. **(I)** and **(J)** Expression of Rps3a in RAW-Control and RAW-Sp110 cells was determined by immunoblotting and qPCR respectively. **(K)** Sp110 promotes Rps3a protein degradation. RAW-Control and RAW-Sp110 cells were treated with protein synthesis Cycloheximide (10 μg/ml) for different periods of time. Expression changes of Rps3a were determined by immunoblotting. **(L)** Overexpression of Sp110 enhances PARP activity. Data are present as means ±SD of three independent experiments, **p< 0.01, and *** p< 0.001.

Ncl is a member of the RNP-containing family of RNA-binding proteins and binds to the 3′ untranslated region (UTR) of some mRNA molecules to enhance their stability [21]. The adenine/uridine-rich elements (ARE) that exist in the 3′ UTR of many mRNAs are one of the most common determinants of RNA stability in mammalian cells [22]. It has been demonstrated that Ncl binds specifically to the ARE in the 3′ UTR of the Bcl2 mRNA protecting it from ribonuclease degradation [21]. Since our data showed that Sp110 interacts with Ncl, we investigated the effect of Sp110 on Ncl and its target Bcl2 mRNA. Remarkably, we found that the level of Ncl protein was decreased significantly in RAW264.7 cells overexpressing Sp110 (Figure 6A). Consequently, both the mRNA and protein products for Bcl2 were also downregulated indirectly by Sp110 (Figure 6A, 6B). Analysis by qPCR showed no significant changes in Ncl mRNA levels between RAW-Sp110 and RAW-Control cells (Figure 6C), indicating that Sp110 inhibits Ncl expression at the post-translational stage. To address the possibility that Sp110 interacts with Ncl protein to promote its degradation, we treated RAW-Sp110 and RAW-Control cells with the protein synthesis inhibitor Cycloheximide (CHX). In RAW-Control cells, the Ncl protein was degraded rapidly following CHX treatment (Figure 6D). Furthermore, treatment with the proteasome inhibitor MG132 prevented this degradation of Ncl (Figure 6D), suggesting that the stability of Ncl protein might be regulated by the proteasome. In contrast, the levels of Ncl protein in RAW-Sp110 cells exhibited significantly more rapid degradation than for the control cells, indicating that the interaction of Sp110 with Ncl promotes its degradation.

To demonstrate that Sp110 inhibits Bcl2 expression indirectly via reducing Ncl protein levels in macrophages, the RAW-Sp110 cells were transfected with an Ncl expression plasmid pCMV-Myc-*Ncl*. We found that overexpression of Ncl successfully rescued the expression of Bcl2 protein compared to the control cells (Figure 6E). Moreover, knockdown of Ncl using siRNAs led to downregulation of both Bcl2 mRNA and protein (Figure 6F, 6G). To confirm that Sp110 inhibits Bcl2 through the reduction of *Bcl2* mRNA stability, we cloned the ARE sequence found in mouse *Bcl2* mRNA and inserted it into the luciferase reporter vector psiCHECK-2 to generate a reporter plasmid psiCHECK2-*Bcl2* ARE. Luciferase assays of RAW264.7 cells cotransfected with either the Sp110 or Ncl expression plasmids, or both, in combination with psiCHECK2-*Bcl2* ARE showed that Sp110 expression inhibits the luciferase activity of psiCHECK2-*Bcl2* ARE. In contrast, overexpression of Ncl enhances the luciferase activity of psiCHECK2-*Bcl2* ARE, and reduces the inhibitory effect of Sp110 (Figure 6H). Together, these results revealed that Sp110 interacts with Ncl protein and promotes its degradation, leading to instability of the Bcl2 mRNA and subsequent decreased expression of Bcl2 protein. This might result in enhancement of apoptosis of macrophages infected with *Mtb*.

Poly (ADP-ribose) polymerase (PARP) is involved in apoptosis since inhibition of PARP activity with PARP inhibitors is known to block apoptosis. In addition, the ribosomal protein Rps3a also inhibits PARP activity via mediating the interaction of Bcl2 and PARP [23]. Since we showed that Sp110 also interacts with Rps3a, we tested the effect of Sp110 on Rps3a. Our results showed that overexpression of Sp110 downregulated the levels of Rps3a protein (Figure 6I). However, the transcription level of Rps3a mRNA showed no significant change in RAW-Sp110 when compared with RAW-Control (Figure 6J), indicating that Sp110 affects protein stability of Rps3a. Inhibition of protein synthesis by CHX confirmed that Sp110 promotes degradation of Rps3a protein (Figure 6K). Moreover, we found that overexpression of Sp110 enhances PARP activity in RAW264.7 cells (Figure 6L). Collectively, these data suggested that Sp110 promotes Rps3a degradation, resulting in upregulation of PARP activity to enhance apoptosis.

### The ER stress response is involved in apoptosis of *Mtb* infected macrophages from Sp110 transgenic cattle

**Figure 7:**
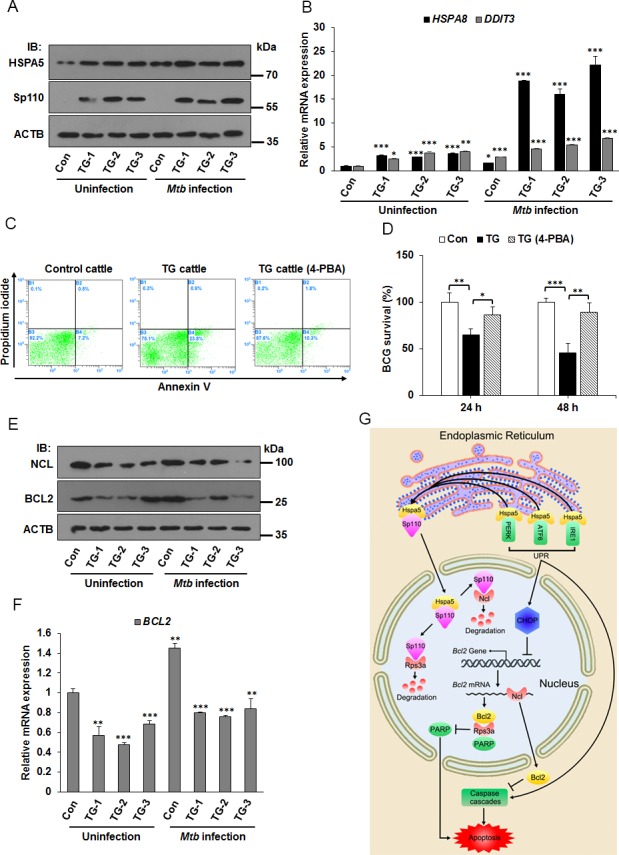
ER stress responses is involved in apoptosis of *Mtb* infected macrophages from Sp110 transgenic cattle **(A)** Sp110 induces ER stress marker Hspa5. Macrophages isolated from control cattle and Sp110 transgenic cattle were infected with BCG (MOI 5:1) for 24 h. The expression of Sp110 and Hspa5 was determined by immunoblots. Con, macrophages from control cattle. TG, macrophages from Sp110 transgenic cattle. **(B)** Total RNA was extracted from macrophages of (A). The expression of *HSPA8* and *DDIT3* was determined by qPCR. **(C)** Macrophages were isolated from control cattle and Sp110 transgenic cattle, and then challenged with BCG (MOI 5:1) for 24 h. The TG cattle (4-PBA) sample was pretreated with 4-PBA for 1 h before *Mtb* infection. 4-PBA remained for the rest of the infection. Cell apoptosis was quantified by Annexin-V staining followed by flow cytometric analysis. **(D)** Macrophages isolated from Sp110 transgenic cattle were pretreated with 4-PBA for 1 h, and then infected with BCG (MOI 5:1) for 24 h and 48 h. 4-PBA remained for the rest of the infection. Intracellular mycobacterial number was determined by CFU counting at the indicated timepoints. **(E)** and **(F)** Sp110 inhibits BCL2 expression in bovine macrophages. Samples were prepared as (A), the protein levels of NCL and BCL2 were detected by immunoblots (E) and the mRNA of *BCL2* was determined by qPCR (F). **(G)** Model of Sp110-mediated macrophage apoptosis. Sp110 competitively binds to Hspa5, which translocates Hspa5 into the nucleus to activate ER stress and initiate the apoptotic pathway. Moreover, Sp110 promotes protein degradation of Ncl and Rps3a, downregulating the anti-apoptotic Bcl2 expression and upregulating PARP activity, thus promoting apoptosis. Data are present as means ±SD of three independent experiments,* p< 0.05, **p< 0.01, and *** p< 0.001.

Transgenic cattle carrying the mouse Sp110 gene were recently created by our team. *In vitro* and *in vivo* challenge with *M. bovis* demonstrated that the transgenic cattle showed lower levels of bacterial growth and multiplication. *M. bovis* infected monocyte-derived macrophages (MDMs) derived from Sp110 transgenic cattle tended to produce apoptosis, whereas infection of MDMs from control cattle produced necrosis [6, 24]. We wondered whether Sp110 could induce the ER stress response in bovine macrophages. To test this hypothesis, the MDMs from control cattle and Sp110 transgenic cattle were isolated, and then challenged with *Mycobacterium bovis* Bacillus Calmette-Guerin (BCG) for 24 h. We found that the protein levels of the ER stress marker Hspa5 increased significantly in macrophages from Sp110 transgenic cattle when compared with those of the control (Figure 7A). Moreover, the downstream genes of ER stress pathway, *HSPA8* and *DDIT3*, were upregulated in macrophages from Sp110 transgenic cattle (Figure 7B). Notably, apoptosis assays revealed that inhibition of the ER stress response blocked apoptosis in BCG infected macrophages derived from Sp110 transgenic cattle (Figure 7C). In addition, we found that treatment with 4-PBA enhanced survival of BCG in macrophages derived from Sp110 transgenic cattle (Figure 7D). These results suggested that Sp110 induces ER stress in bovine macrophages, and this is crucial for Sp110-enhanced resistance of bovine macrophages to BCG infection.

Next, we asked whether Sp110 inhibits Ncl and Bcl2 expression in bovine macrophages. As expected, the immunoblots showed that levels of both NCL and BCL2 proteins were decreased in macrophages derived from Sp110 transgenic cattle (Figure 7E). Furthermore, the mRNA of *BCL2* in macrophages from Sp110 transgenic cattle was lower than that from control cattle (Figure 7F). Taken together, our data suggest that Sp110 interacts with Hspa5 to activate the ER stress response and that the downstream UPR is essential for Sp110-enhanced apoptosis in macrophages following *Mtb* infection. Moreover, Sp110 promotes Ncl protein degradation, subsequently downregulating the protein level of the anti-apoptotic Bcl2. Furthermore, Sp110 promotes Rps3a degradation to enhance the activity of pro-apoptotic PARP, which also contributes to the activation of the apoptotic pathway (Figure 7G).

## DISCUSSION

Previous studies have investigated the significance of certain genes in regulating innate immunity to intracellular pathogen, and identified the role of mouse Sp110 in the process of *Mtb* infection [5, 7, 25–27]. To date, however, the molecular mechanisms by which Sp110 executes these physiological functions remain unknown. Here, we investigated the Sp110 protein interactome using transgenic mouse macrophages expressing a biotinylated version of Sp110. In addition to the validation of previously characterized Sp110-interacting proteins Hspa8 [12] and Mybb1pa [13], this analysis identified 251 new Sp110-interacting proteins, this supports the conclusion that the biotin-streptavidin based approach using biotin tagged Sp110 is a reliable and robust method to identify proteins associated with Sp110.

We, and others, have demonstrated the critical role of Sp110 in switching on the apoptotic pathway in *Mtb*-infected macrophages [5, 7]. The Sp110 interactome revealed that Sp110 does not interact with the apoptotic effectors directly, but interacts with multiple apoptotic regulators, including Ncl, Bag1, Bag2, Bag5, Vcp, and Wwox [28–33]. The interaction between Sp110 and Ncl, Bag2, Vcp, or Wwox was validated by IP, suggesting that Sp110 binds to such partners and modulates their amounts or conformations to alter cell death pathway. Furthermore, we identified and validated that Sp110 interacts with the ER stress marker Hspa5. Considering the importance of Hspa5 in ER stress-induced apoptosis [18], we speculate that Sp110 may regulate the ER stress response upon *Mtb* infection and thus determine whether infected macrophages survive or undergo apoptosis. In addition, we noted that the largest group of Sp110-interacting proteins was implicated in translation and ribosome biogenesis, indicating that Sp110 may be involved in some unexplored functions, and idea confirmed by a recent study [34].

Inhibition of apoptosis is a strategy for virulent *Mtb* to evade host immune responses, and as a result, the main functions of Sp110 in *Mtb* infected host cells is reactivating the apoptotic pathway [5, 7]. The Sp110 interactome data suggest that Sp110 influences the ER stress response, inducing its activation via sequestering Hspa5 in the nucleus through protein-protein interaction. Moreover, inhibition of the ER stress response using chemicals significantly decreased Sp110-enhanced apoptosis and increased intracellular survival of *Mtb* in both mouse and bovine macrophages. We identified the SAND domain, a conserved ∼80 residue region found in a number of nuclear proteins [35], as the region of Sp110 required for Hspa5 nuclear accumulation and Sp110-enhanced macrophages apoptosis. These results indicate that activation of ER stress-induced apoptosis is the main mechanism for the reactivation of apoptosis by Sp110.

Increased P-eIF2α and CHOP are hallmarks of UPR activation in response to ER stress [36], and were confirmed as being increased in RAW264.7 cells that expressed Sp110 transiently. However, RAW264.7 cells stably expressing Sp110 showed lower levels of P-eIF2α and CHOP than control cells. Previous studies showed that long-term mild ER stress activated all UPR sensors, but survival was favored during mild stress because of the intrinsic instabilities of the mRNAs and proteins of proapoptotic genes (CHOP) compared with those that facilitate protein folding and adaptation (Hspa5) [19]. We speculate that macrophages that were stably expressing Sp110 adapted to ER stress because induction of the UPR pathways led to the death of highly activated cells during the screening process. ER stress-adapted cells undergo CHOP-induced apoptosis when subjected to even low-level ER stress [36] (such as *Mtb* infection caused ER stress), which explains the similarity of the RAW-Sp110 apoptotic rates to the RAW-Control rates before *Mtb* infection but significantly higher rates after *Mtb* infection.

The anti-apoptotic protein Bcl2 inhibited apoptotic death by preventing cytochrome c release from the mitochondrial intermembrane space, and further inhibiting the activation of caspase proteases [37]. Post-translational regulation of Bcl2 mRNA is thought to be a critical factor for controlling Bcl2 protein expression. In particular, the binding of the RNA binding protein Ncl to the AREs present in the 3′ UTR of the *Bcl2* mRNA has been functionally implicated in stabilizing the *Bcl2* mRNA [21, 38]. We found that Sp110 interacts with Ncl and promotes Ncl degradation, thus diminishing *Bcl2* mRNA stability and, as a result, protein levels in RAW264.7 cells. PARP was suggested as a proapoptotic protein because inhibition of PARP activity blocks apoptosis [39]. A previous study suggested that Rps3a represses PARP activity [23], which is consistent with our data that revealed that Sp110 interacts with Rps3a and promotes Rps3a protein degradation, thus upregulating PARP activity. These results showed alternative mechanisms of Sp110 to enhance apoptosis. Importantly, the results of experiment on macrophages isolated from Sp110 transgenic cattle support the conclusion that Sp110 activates ER stress-induced apoptosis and inhibits Bcl2 expression.

In summary, we used a streptavidin-mediated affinity purification approach to comprehensively analyze the composition of the Sp110 interactome in murine macrophages, and identified many previously uncharacterized Sp110 associated proteins which were subsequently validated. Our results revealed that the interaction between Sp110 and Hspa5 is essential for activating ER stress-mediated apoptosis in both mouse and bovine macrophages. In addition, we found that Sp110 is able to decrease the stability of Ncl and Rps3a proteins, thereby enhancing apoptosis. These findings have extended our understanding of the mechanism of Sp110-mediated apoptosis and macrophage resistance to *Mtb*, and provided data for application of Sp110 in tuberculosis-resistant animal breeding program.

## MATERIALS AND METHODS

### Ethics statement

This study was performed in strict accordance with the guidelines for the care and use of animals of Northwest A&F University. The animal experiments were approved by the Animal Care Commission of the College of Veterinary Medicine, Northwest A&F University. Every effort was made to minimize animal pain, suffering, and distress and to reduce the number of animals used.

### Monocyte extraction and culture of bovine MDMs

The MDMs of control cattle and Sp110 transgenic cattle were isolated as described previously [40]. Briefly, 100 ml blood collected from control cattle and transgenic cattle were transferred into 50 ml centrifuge tubes containing acid citrate dextrose buffer (Sigma-Aldrich). Blood was layered by centrifugation over histopaque-1077 (Sigma-Aldrich). MDMs were collected and seeded into 10 cm dishes containing RPMI1640 medium supplemented with 10% FBS for 2 h at 37°C, 5% CO2. Non-adherent cells were removed by washing with PBS. Adherent cells were maintained in culture and differentiated into macrophages after 5 days, confirmed by morphology and Giemsa staining.

### Cell culture and *Mtb* infection

RAW264.7 cells purchased from the American Type Culture Collection (ATCC) were maintained in RPMI1640 medium containing 10% FBS. HEK293T cells (ATCC) were cultured in DMEM supplemented with 10% FBS. All cells were maintained at 37°C and 5% CO2 in a humidified incubator. *M. tuberculosis* strain H37Ra (ATCC 25177) and *M. bovis* Bacillus Calmette-Guerin (BCG) Danish strain 1331 (NIBSC, Hertfordshire, UK) were maintained in Middlebrook 7H9 broth medium or on 7H10 agar plates supplemented with 10% OADC (Becton, Dickinson and Company, Franklin Lakes, NJ) and cultured in a tissue culture incubator at 37°C with 5% CO2 and 95% air. The cells were infected for 4 h with *Mtb* H37Ra or BCG at a MOI of 5:1, then cells were washed with PBS to remove extracellular bacteria and cultured with fresh complete medium. Intracellular bacterial counts were determined at the indicated times after infection on Middlebrook 7H10 agar plates.

### Generation of constructs and stably transfected cells

The *Sp110* coding sequence was amplified by PCR using cDNA from C57BL/6 mouse lung tissue. The coding sequence of biotin ligase *BirA* was amplified from genomic DNA of Escherichia coli BL21 (DE3). For constructing the lentiviral constructs BAP-Sp110-P2A-BirA-T2A-PuroR, BAP-Sp110-T2A-PuroR and BAP-P2A-BirA-T2A-PuroR, the expression cassettes containing FLAG tag (MDYKDDDDK), Myc tag, BAP (MAGLNDIFEAQKIEWHE), P2A (GSGATNFSL-LKQAGDVEENPGP), *Sp110* and *BirA* ORF sequence were assembled according to their relative location by overlap extension PCR (Figure 1A), and then cloned into the lentiviral expression vector pCDH-MCS-T2A-Puro-MSCV (System Biosciences, Mountain View, CA) between NheI and NotI. The lentiviral expression vector pCDH-Sp110 was generated by inserting Sp110 ORF sequence into the pCDH-MCS-T2A-Puro-MSCV vector.

A Flag-tagged *Sp110* ORF sequence was inserted into the pCDNA3.1 vector to generate the expression plasmid pCDNA3.1-FLAG-*Sp110*, and pCMV-Myc-*Ncl* was constructed by inserting the ORF sequence of *Ncl* gene into the pCMV-Myc vector. The ORF sequence of *Hspa5* gene was cloned into the pCMV-HA vector to generate the expression vector pCMV-HA-*Hspa5*. To generate the reporter plasmid psiCHECK2-*Bcl2* ARE, 300 bp of the mouse Bcl2 3′ UTR sequence rich in AREs was amplified by RT-PCR, and then cloned into psiCHECK-2 vector (Promega, Madison, WI).

To generate lentiviruses transduced cells, RAW264.7 cells were transduced with viral supernatants collected from the HEK293T cells transfected with lentiviral constructs BAP-Sp110-P2A-BirA-T2A-PuroR, BAP-Sp110-T2A-PuroR, BAP-P2A-BirA-T2A-PuroR, pCDH-Sp110 and empty vector pCDH-MCS-T2A-Puro-MSCV respectively. After 48 h transduction, puromycin (5 μg/ml) was added to the dishes for additional 5 d to screen for stably transfected cells, and expression of target genes was verified by western blotting.

### Nuclear extract preparation and Sp110 complexes purification

Nuclear extracts of RAW-BAP-Sp110-P2A-BirA cells and RAW-BAP-P2A-BirA cells (2×10^9^ cells each) were prepared as described [41]. The protein concentration of nuclear extracts was quantified using BCA Protein Assay Reagent (Pierce, Rockford, IL), equal amounts of nuclear extract protein from RAW-BAP-Sp110-P2A-BirA cells and RAW-BAP-P2A-BirA cells were used. For the purification of Sp110 protein complexes, nuclear extracts were diluted using an equal volume of cold buffer 1 (350 mM NaCl, 20 mM Tris-HCl (pH 7.5), 0.5% NP-40 (vol/vol), 1 mM EDTA and 10% glycerol (vol/vol)) supplemented with DTT (1 mM), 1 mM PMSF (1 mM) and protease inhibitor cocktail (#88266, Pierce), and precleared by incubation with protein G-agarose (Pierce) for 1-2 h at 4°C while rotating. Samples were centrifuged for 5 min at 300 g and then the precleared supernatants transferred to new tubes and incubated with streptavidin-conjugated Agarose resin (#20357, Pierce) overnight at 4°C with rotation. The beads were washed 4 times (15 min per wash) with buffer 2 (350 mM NaCl, 20 mM Tris-HCl (pH 7.5), 0.3% NP-40 (vol/vol), 1 mM EDTA and 10% glycerol (vol/vol), supplemented with DTT (1 mM), 1 mM PMSF (1 mM) and protease inhibitor cocktail), and then resuspended in 2× reducing laemmli buffer followed by boiling for 5 min at 98°C.

### Mass-spectrometric (MS) analysis of Sp110 complexes

Protein samples from the streptavidin-mediated affinity purification were separated by 4–20% gradient SDS-PAGE (Bio-Rad), stained with Acqua stain protein gel dye (Bulldog Bio Inc., Portsmouth, NH), bands were excised from the gel lanes and digested with trypsin. MS was performed on a TripleTOF 5600 System (AB SCIEX, Concord, ON) by BGI (Shenzhen, Guangdong, China), detailed procedures have been described previously [42]. Proteins were identified using Mascot software (version 2.3.02) using the IPI_Mouse database (59534seqs).

### Immunoprecipitation (IP) and immunoblotting (IB)

Nuclear lysates of cells were extracted using a Nuclear and Cytoplasmic Extraction Kit (Pierce) according to the manufacturer's instructions. The protein concentration of nuclear lysates were quantified using BCA Protein Assay Reagent (Pierce), and then an equal amount of protein for each sample was incubated with streptavidin-conjugated agarose resin (Pierce) at 4°C overnight. Beads were washed thoroughly with IP350 (0.3%) buffer and resuspended in reducing laemmli loading buffer, and boiled for 5 minutes at 98°C. The protein samples were separated by SDS-PAGE followed by western blotting analysis. Primary antibodies used were Hspa8 (1966-1), Pcna (2714-1), Rps3a (7944-1), Ybx1 (2397-1), Prdx1 (3688-1), Bag2 (2622-1) purchased from Epitomics (Burlingame, CA), Wwox (4045S), Eif4g2 (5169P), Mcm3 (4012S), Myc (2276S), Casp3 (9662S), Eif2α (9722S), P-Eif2α (9721S), Vav1 (2502S), CHOP (5554S) and Anxa2 (8235S) purchased from Cell Signaling Technology (Danvers, MA), Oasl1 (sc-98385), Hspa5 (sc-13968), Sp110 (sc-98365), Ncl (sc-13057), and Bcl2 (sc-492) purchased from Santa Cruz Biotechnology, Inc. (Dallas, TX), FLAG (F1804-200ug, Sigma, Saint Louis, MO), Vcp (Ab109240, Abcam Cambridge, MA), Atp2a2 (AB54032-25ul, Sangon, Shanghai, China), and Actin (TransGen Biotech Co., Ltd, Beijing, China). Secondary antibodies were purchased from Beyotime (Beyotime, Jiangsu, China).

### Immunofluorescence staining

All buffers used in immunofluorescence staining were purchased from Beyotime Institute of Biotechnology (Nanjing, Jiangsu, China). Cells were fixed and permeabilized using immunostaining fixation buffer for 20 min, and then blocked in blocking buffer for 2 h. The indicated primary antibody was added to the plates overnight at 4°C, followed by three washes with washing buffer for 5 min and then incubation with an Alexa Fluor 488/555-conjugated secondary antibody for 2 h at room temperature. Nuclei were stained with DAPI. Cells were photographed under an inverted fluorescence microscope (Nikon, Tokyo, Japan).

### Reverse transcription (RT) and quantitative real-time PCR (qPCR)

Total RNA was isolated using Trizol reagent (Invitrogen). First-strand cDNA was synthesized using a SYBR PrimeScript RT reagent Kit (Perfect Real Time) (Takara, Dalian, China). The qPCR was performed using SYBR Premix Ex Taq II (Tli RNaseH Plus) (Takara) on a StepOnePlus PCR system (Applied Biosystems, Foster City, CA). The procedures for the qPCR were as follows: 95°C for 1 min, followed by 40 cycles of 95°C for 5 sec and 60°C for 30 sec. The specificity of the primer amplicons was examined by the analysis of a melting curve. The comparative Ct method was employed for quantification of target mRNA expression that was normalized to *Gapdh* expression and relative to the calibrator. The primers used were provided in Supplementary Table 4.

### Luciferase reporter assays

RAW264.7 cells were cultured in 24-well plates one day prior to transfection, and then cotransfected with the luciferase reporter plasmid psiCHECK2-*Bcl2* ARE and pcDNA3.1-Flag-*Sp110* or pCMV-Myc-*Ncl*. Cells were harvested 48 h later, and luciferase activity was detected using the dual-luciferase reporter assay system (Promega) following the manufacturer's protocol.

### Transient transfection

RAW264.7 cells were transfected with siRNAs targeting mouse *Ncl* (si-Ncl) or negative control (si-NC) (Life Technologies, Shanghai, China) using Lipofectamine 2000 (Invitrogen, Carlsbad, CA). The target sequences of siRNA were as follows, si-Ncl-1: GCAAGGATCCAATTCGAGA, si-Ncl-2: CAACTACACCTTT CAATCT. HEK293T or RAW264.7 cells were transfected with plasmid (0.5 μg/well for 24 well plate) using FuGENEHD reagent (Promega) according to the manufacturer's instructions.

### Detection of nascent protein synthesis

Click-iT HPG Alexa Fluor® 488 Protein Synthesis Assay Kit (Molecular Probes, Eugene, OR) was used to label newly synthesized protein. This kit includes an amino acid analog of methionine L-homopropargylglycine (HPG) containing an alkyne moiety. The HPG is incorporated into proteins during active protein synthesis. Addition of the Alexa Fluor® 488 azide leads to a chemoselective ligation between the green fluorescent azide and the alkyne, allowing the modified proteins to be detected by imaged-based analysis. Briefly, Cells were plated on 24-well plates and treated as indicated, and then labeled in methionine-free media containing 50 μM Click-iT HPG for 40 min, and subsequently fixed and permeabilized followed by immunofluorescence and or Click-iT HPG detection.

### Apoptosis analysis

Apoptotic cells were assessed by binding of Annexin V-Alexa Fluor 488 (Molecular Probes, Eugene, OR). Cells were incubated in Annexin binding buffer (10 mM HEPES, 140 mM NaCl and 2.5 mM CaCl2) and stained with 10 μl Annexin V-Alexa Fluor 488 for 20 min, followed by counterstaining with propidium iodide (PI, 1 μg/ml) for 15 min. Finally, cells were washed twice with cold PBS and fixed with 1% paraformaldehyde for 30 min, and then washed once with cold PBS. Binding of Annexin V-Alexa Fluor 488 and PI was analyzed by FACS Calibur flow cytometer (BD Biosciences, San Jose, CA).

### PAPR activity assay

PARP activity was examined using a universal colorimetric PARP assay kit (#4677-096-K, R&D Systems, Minneapolis, MN), the standard curve and sample assay were conducted following the manufacturer's instructions.

### Statistical analysis

The data were represented as the mean ± SD and were analyzed using the Student's t-test. A value of p<0.05 was considered significant. Data were presented as mean ± standard deviation from at least three independently experiments.

## SUPPLEMENTARY MATERIALS FIGURE AND TABLES


