# Supplementary material for: Sp110 enhances macrophage resistance to Mycobacterium tuberculosis via inducing endoplasmic reticulum stress and inhibiting anti-apoptotic factors
Source: Oncotarget. 2017 Jul 17;8(38):64050–65. doi: 10.18632/oncotarget.19300 (PMC5609983; doi:10.18632/oncotarget.19300)
Supplement: Supplementary file 1 [file oncotarget-08-64050-s001.pdf]

Sp110 enhances macrophage resistance to *Mycobacterium tuberculosis* via inducing endoplasmic reticulum stress and inhibiting anti-apoptotic factors

SUPPLEMENTARY MATERIALS

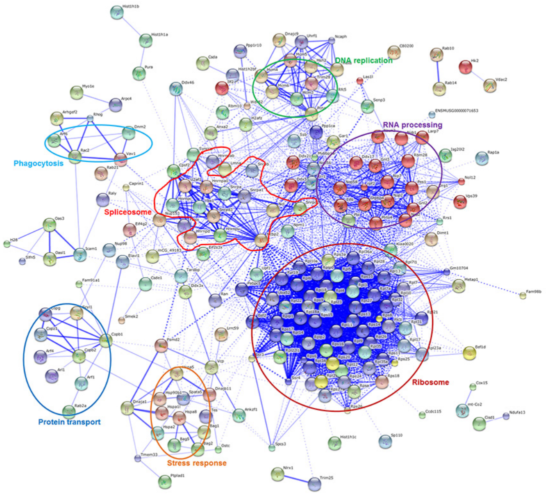

Supplementary Figure 1. Network map of the known interactions among Sp110-interacting proteins. The protein-protein interaction network was generated by STRING 10 software package. The known protein complexes are depicted as colored circles**.**

Supplementary Table 1: Ipr1 interactome identified by mass spectrometry.

See Supplementary File 1

Supplementary Table 2: KEGG pathway analysis of Ipr1 interacting proteins.

See Supplementary File 2

Supplementary Table 3: Mass spectrometry identified Ipr1-interacting partners which involved in ribosome biogenesis.

See Supplementary File 3

Supplementary Table 4: Primers used for qPCR analysis of mRNA

Primer name	Sequence (5’-3’)
qBcl2-F (m)	GGAGGCTGGGATGCCTTTGT
qBcl2-R (m)	TGCACCCAGAGTGATGCAG
qNcl-F(m)	CGCCTTTCCAGAGGCGATTA
qNcl-R(m)	GTGGGTTTTGCCAGCCTTTG
qRps3a-F(m)	GGTCAAGAAGTGGCAGACCA
qRps3a-R(m)	TGCTGCGCATAGGATGTCTT
qGapdh-F (m)	GTGTTCCTACCCCCAATGTGT
qGapdh-R (m)	ATTGTCATACCAGGAAATGAGCTT
qHSPA5-F (B)	CCTGTTCCGTTCCACCATGA
qHSPA5-R (B)	CCACAGCCTCATCTGGGTTT
qDDIT3-F (B)	CACCTGAAAGCAGAGCCTGA
qDDIT3-R (B)	ACAGGTGCCCCGATTTTCAT
qHSPA8-F (B)	TGGCAGGCCTAAGGTTCAAG
qHSPA8-R (B)	AGCGTTGGTAACCGTCTTCC
qBCL2-F (B)	TCATGTGTGTGGAGAGCGTC
qBCL2-R (B)	CTCCACAAAGGCGTCCCAG
qGAPDH-F (B)	GGCGTGAACCACGAGAAGTA
qGAPDH-R (B)	GGCGTGGACAGTGGTCATAA

m: mouse; B: Bovine
